# Stationary Wavelet-Fourier Entropy and Kernel Extreme Learning for Bearing Multi-Fault Diagnosis

**DOI:** 10.3390/e21060540

**Published:** 2019-05-28

**Authors:** Nibaldo Rodriguez, Lida Barba, Pablo Alvarez, Guillermo Cabrera-Guerrero

**Affiliations:** 1 Escuela de Ingeniería Informática, Pontificia Universidad Católica de Valparaíso, Valparaíso 2374631, Chile; 2 Facultad de Ingeniería, Universidad Nacional de Chimborazo, Chimborazo 060108, Ecuador

**Keywords:** stationary wavelet packet transform, multi-scale entropy, Fourier amplitude spectrum kernel extreme learning machine

## Abstract

Bearing fault diagnosis methods play an important role in rotating machine health monitoring. In recent years, various intelligent fault diagnosis methods have been proposed, which are mainly based on the features extraction method combined with either shallow or deep learning methods. During the last few years, Shannon entropy features have been widely used in machine health monitoring, improving the accuracy of the bearing fault diagnosis process. Therefore, in this paper, we consider the combination of multi-scale stationary wavelet packet analysis with the Fourier amplitude spectrum to obtain a new discriminative Shannon entropy feature that we call stationary wavelet packet Fourier entropy (SWPFE). Features extracted by our SWPFE method are then passed onto a shallow kernel extreme learning machine (KELM) classifier to diagnose bearing failure types with different severities. The proposed method was applied on two experimental vibration signal databases of a rolling element bearing and compared to two recently proposed methods called stationary wavelet packet permutation entropy (SWPPE) and stationary wavelet packet dispersion entropy (SWPPE). Based on our results, we can say that the proposed method is able to achieve better accuracy levels than both the SWPPE and SWPDE methods using fewer failure features. Further, as our method does not require any hyperparameter calibration step, it is less dependent on user experience/expertise.

## 1. Introduction

Health conditions of rolling-element bearings (REBs) play a vital role in the working performance of the rotation machine. Therefore, REBs’ fault diagnosis is a very important task to guarantee the availability and reliability of the rotation machines in industrial processes. In recent years, quite a few intelligent fault diagnosis approaches have been proposed, which are mainly based on features extraction methods combined with either shallow or deep learning methods. Moreover, during the last few years, several bearing health indicators such as Shannon entropy, spectral kurtosis (SK), the smoothness index [1], the Gini index [2,3], and the spectral Lp/Lq norm [4,5] have been used in bearing failure diagnosis. Besides vibration signals, other signals such as current signals [6,7], acoustic signals [8], and stray flux signals [9] have also been used for fault diagnosis. In this paper, we use vibration signals as they are easier to measure and can provide useful dynamic information that reflects bearings’ health condition.

In particular, the SK helps to determine the location of which frequency bands are more informative according to their impulsivity (maximum kurtosis value). Various studies related to SK have been presented over the last decade. Antoni [10] proposed an SK estimation method based on the short-time Fourier transform. Antoni and Randall [11] published a diagnosis method called the kurtogram, which shows SK values as a function of both the center frequency and bandwidth of the filtered signal. However, it has been shown that the kurtogram is computationally expensive, and thus, the fast kurtogram was proposed by Antoni [12]. Since then, several diagnosis methods based on improved spectral kurtosis have been reported by other researchers [13,14,15,16,17]. Wang [5] showed that the reciprocal of the spectral smoothness index and spectral Gini index was less sensitive to outliers than spectral kurtosis and spectral L2/L1 norm. Therefore, bearing health indicators based on both the reciprocal of the spectral smoothness index and the spectral Gini index are more appropriate for the fault features extraction process. Although all these indicators have been proven to be very effective in early bearing fault detection, in this paper, we are focused on the time-frequency Shannon entropy as it has been shown to be a more informative failure feature.

Since the raw vibration signals recorded from REBs are in the time domain, we need to transform it into the time-frequency domain by using some appropriate transformation method. The time-frequency domain features for REBs fault diagnosis can be extracted using different failure features extraction methods. For instance, in [18], empirical mode decomposition (EMD) was combined with improved frequency band entropy in bearing fault feature extraction. Han and Pan [19] used local mode decomposition (LMD) combined with sample entropy and the energy ration to improve the fault diagnosis in REBs. Gong et al. [20] used variational mode decomposition, and in Rodriguez et al. [21], wavelet transform was combined with dispersion entropy and also with permutation entropy, which in turn was passed onto a kernel extreme learning machine classifier. Unlike in [21], in Rodriguez et al. [22], the feature extraction method was based on the stationary wavelet transform and a singular value decomposition. Wavelet transform has also been used in combination with the conventional statistical index and the logarithmic energy entropy [23]. After the effective features have been selected, all the methods mentioned above pass the extracted features to a machine learning classifier. Traditionally, shallow learning classifiers such as the artificial neural network [24] and support vector machine [25] have been used, and more recently, deep learning classifiers such as deep output kernel learning [26] and the convolutional neural network [27,28,29] have also been used.

Based on these studies, including extensions of the Shannon entropy seems to be an efficient strategy to improve the accuracy of the bearing fault diagnosis. Thus, in this article, we consider transforming the time-frequency Shannon entropy into a frequency domain Shannon entropy to obtain better quality failure features. To this end, we propose to integrate stationary wavelet packet (SWP) transform with Fourier amplitude spectrum, leading to what we call stationary wavelet packet Fourier entropy (SWPFE).

After the entropy features extraction, a KELM classifier is used to perform automatic fault diagnosis. The KELM classifier is created by replacing the ELM’s hidden activation function with a Gaussian kernel function and so to improve the generalization performance of ELM and reduce time consumption for determining the number of hidden layer nodes [30,31,32]. We chose to use KELM as it has been shown to be very efficient in both classification accuracy and tuning time. In particular, as demonstrated by Huang et al. [31], the KELM classifier outperforms other well-known classification methods such as support vector machines and least-square support vector machines.

We apply our diagnosis method on two bearing vibration signals databases under variable work conditions obtained in [33,34], namely fan-end and drive-end datasets. Using these datasets, a comparison among the accuracy obtained by our SWPFE method and the results obtained in [21,22] is performed. We need to point out that, unlike the vast majority of the studies that have been working on these datasets, we decided to consider the drive-end dataset of 48 kHz rather than the drive-end dataset of 12 kHz. We do this because the drive-end dataset of 48 kHz is much harder to tackle, as stated in [26], and thus, it allows us to clearly show the difference in the accuracy levels obtained by our proposed method when compared to other state-of-the-art bearing failure diagnosis algorithms.

This work is organized as follows: in Section 2, we briefly present three measures of time-frequency Shannon entropies and the SWP transform. In Section 3, we describe the bearing multi-fault diagnosis algorithm implemented in this paper and the setup we consider for our experiments. In Section 4, we analyze the results obtained by our algorithms. We draw some conclusions in Section 5.

## 2. Shannon Entropy Measures

In this section, we introduce three Shannon entropy measures, namely SWPFE, SWPPE, and SWPDE, as in [21].

### 2.1. Stationary Wavelet Packet Fourier Entropy

Let w(n) be a signal corresponding to one of the $D=2^{J}$ stationary wavelet sub-band signals (see Equations (13a) and (13b)), then its stationary wavelet packet Fourier entropy (SWPFE) is calculated through the following steps:

(1a) $$\begin{array}{cc} E & =-\frac{1}{log_{2}\left(K\right)}\sum_{k=1}^{K}p\left(k\right)log_{2}\left(p\left(k\right)\right)\;K=\frac{2^{a}}{2},\;a=\left\lceil log_{2}\left(N\right)\right\rceil \end{array}$$

(1b) $$\begin{array}{cc} p(k) & =\frac{s^{2}\left(k\right)}{\sum_{k=1}^{K}s^{2}\left(k\right)} \end{array}$$

where s(k) represents the amplitude spectrum obtained as follows:

(2a) $$\begin{array}{cc} s(k) & =\sqrt{ℜ{\left[X\left(k\right)\right]}^{2}+ℑ{\left[X\left(k\right)\right]}^{2}} \end{array}$$

(2b) $$\begin{array}{cc} X(k) & =\sum_{n=0}^{N}\sqrt{w(n)}exp\left(-j2\pi kn/N\right)\;k=0,\ldots ,K-1 \end{array}$$

where X(k) denotes the $k^{\mathrm{th}}$ Fourier coefficient in the frequency domain, $N_{1}$ is the size of the Fourier series, and *ℜ* and *ℑ* represent the real and the imaginary parts of the complex spectrum X(k), respectively.

### 2.2. Stationary Wavelet Packet Permutation Entropy

The stationary wavelet packet permutation entropy (SWPPE) of a wavelet sub-band signal $\left\{w\left(n\right)=w_{i}\left(n\right),i=2^{J},n=1,\ldots ,N\right\}$ obtained by using Equations (13a) and (13b) is calculated through the following steps [35]:

*Step* *1:* Create a set of *m*-dimensional vectors $W_{i}^{m}$ as follows:
  (3) $$W_{i}^{m}=\left[w\left(i\right),w\left(i+1\right),\ldots ,w\left(i+m-1\right)\right],\;i=1,2,\ldots ,N-m+1$$
  where *m* is the embedding dimension of the vector $W_{i}^{m}$.
*Step* *2:* Each vector $W_{i}^{m}$ is sorted in ascending order with permutation pattern π as follows:
  (4a) $$\begin{array}{cc} W_{i}^{m} & =[w\left(i+j_{1}-1\right)\leq w\left(i+j_{2}-1\right),\leq \ldots \leq ,w\left(i+j_{m}-1\right)] \end{array}$$
  (4b) $$\begin{array}{cc} \pi & =[j_{1},j_{2},\ldots ,j_{m}] \end{array}$$
  where each vector $W_{i}^{m}$ in *m*-dimensional space can be mapped to one of the m! ordinal patterns π.
*Step* *3:* Calculate the probability of occurrence for each permutation pattern π as follows:
  (5) $$p\left(\pi \right)=\frac{\mathrm{Number}\left\{i|i=1,2,\ldots ,N-m+1;W_{i}^{m}\;\mathrm{has}\;\mathrm{type}\;\pi \right\}}{N-m+1}$$
  where N−m+1 denotes the total amount of embedding vectors.
*Step* *4:* Calculate the normalized SWPPE of the $i^{\mathrm{th}}$ wavelet sub-band signal w(n) using Equation (6):
  (6) $$SWPPE\left[w\left(n\right)\right]=\frac{-1}{log\left(m!\right)}\sum_{j=1}^{m!}p\left(\pi_{j}\right)log\left(p\left(\pi_{j}\right)\right)$$
  Here, for all the experimental examples, the embedding dimension is selected as m=6 [21,35].

### 2.3. Stationary Wavelet Packet Dispersion Entropy

Let w(n) be a signal corresponding to one of the $D=2^{J}$ wavelet sub-band components, then its stationary wavelet packet dispersion entropy (SWPDE) is calculated through the following steps [36]:

*Step* *1:* The wavelet sub-band signal {w(n)} is normalized between zero and one using the normal cumulative distribution function as follows:
  (7) $$y\left(n\right)=\frac{1}{\sigma \sqrt{2\pi }}\int_{-\infty }^{w(n)}exp\left[\frac{-{\left(t-\mu \right)}^{2}}{2\sigma^{2}}\right]dt$$
  where μ and σ are the mean and standard deviation of the raw vibration signal of *N* data points.
*Step* *2:* The normalized signal y(n) is mapped into *c* classes with integer indices from 1–*c* using the equation as follows:
  (8) $$z^{c}\left(n\right)=round\left(c\cdot y(n)+0.5\right)\;n=1,2,\ldots ,N$$
  where round(·) denotes the rounding operation.
*Step* *3:* Create multiple *m*-dimensional vectors $z_{i}^{c,m}$ as follows:
  (9) $$z_{i}^{c,m}=\left[z^{c}\left(i\right),z^{c}\left(i+1\right),\ldots ,z^{c}\left(i+m-1\right)\right],\;i=1,2,\ldots ,N-m+1$$
*Step* *4:* Each embedding vector $z_{i}^{c,m}$ is mapped into a dispersion pattern $\pi_{v_{0},v_{1},\ldots ,v_{m-1}}$, where $z^{c}\left(i\right)=v_{0},z^{c}\left(i+1\right)=v_{1},\ldots ,z^{c}(i+\left(m-1\right)=v_{m-1}$. Thus, the number of possible dispersion patterns is equal to $c^{m}$.
*Step* *5:* Calculate the probability of occurrence for each permutation pattern $\pi_{v_{0},v_{1},\ldots ,v_{m-1}}$ as follows:
  (10) $$p\left(\pi_{v_{0},v_{1},\ldots ,v_{m-1}}\right)=\frac{\mathrm{Number}\left\{i|i=1,2,\ldots ,N-m+1;z_{i}^{c,m}\;\mathrm{has}\;\mathrm{type}\;\pi_{v_{0},v_{1},\ldots ,v_{m-1}}\right\}}{N-m+1}$$
  where N−m+1 denotes the total of embedding vectors.
*Step* *6:* Calculate the normalized SWPDE of the $i^{\mathrm{th}}$ wavelet sub-band signal w(n) using Equation (11):
  (11) $$SWPDE\left[w\left(n\right)\right]=\frac{-1}{log\left(c^{m}\right)}\sum_{\pi =1}^{c^{m}}p\left(\pi_{v_{0},v_{1},\ldots ,v_{m-1}}\right)log\left(p(\pi_{v_{0},v_{1},\ldots ,v_{m-1}})\right)$$
  Here, for all the experimental examples, the embedding dimension is set to m=2 and the number of classes selected as c=5 [21,36,37,38,39].

### 2.4. Stationary Wavelet Packet Transform

The SWPT is similar to both the stationary wavelet transform [40,41,42] and the discrete wavelet transform (DWT) [43,44]. At the first level of wavelet decomposition, an input signal $\left\{x\left(n\right)=w_{0,0}\left(n\right),n=1,\ldots ,N\right\}$ is convolved with a low-pass filter $h_{1}$ defined by a sequence $h_{1}\left(n\right)$ of length *r* and a high-pass filter $g_{1}$ defined by a sequence $g_{1}\left(n\right)$ of length *r*. Both the approximation coefficient $w_{1,1}$ and the detail coefficient $w_{1,2}$ are obtained as follows:

(12a) $$\begin{array}{cc} w_{1,1}\left(n\right) & =\sum_{k=0}^{r-1}h_{1}\left(k\right)w_{0,0}\left(n-k\right) \end{array}$$

(12b) $$\begin{array}{cc} w_{1,2}\left(n\right) & =\sum_{k=0}^{r-1}g_{1}\left(k\right)w_{0,0}\left(n-k\right) \end{array}$$

Since no sub-sampling is performed, the obtained sub-bands signals $w_{1,1}\left(n\right)$ and $w_{1,2}\left(n\right)$ have the same number of elements as the input signal $w_{0,0}\left(n\right)$. Filters $h_{j}$ and $g_{j}$ are computed by using an operator called dyadic up-sampling. Using this operator, zero values are inserted between each pair of elements in the filter that are adjacent. Thus, the SWPT is defined by the pair of filters (low- and high-pass filters) that is chosen and the number of decomposition steps *J*. For this paper, a pair of Db2 wavelet filters has been chosen [34,43]. In the literature, wave filters with order greater than two have also been proposed [23]. Although wave filters with order greater than two have better discriminatory potential both in the time and frequency domain [23], we found that increasing the order of the wave filters does not lead to better diagnosis accuracy levels. Thus, we chose to use the simplest mother wavelet filter, i.e., Db2.

The general process of the SWPT is continued recursively for j=2,…,J as follows:

(13a) $$\begin{array}{cc} w_{j,2i-1}\left(n\right) & =\sum_{k=0}^{r-1}h_{j}\left(k\right)w_{j-1,i}\left(n-k\right) \end{array}$$

(13b) $$\begin{array}{cc} w_{j,2i}\left(n\right) & =\sum_{k=0}^{r-1}g_{j}\left(k\right)w_{j-1,i}\left(n-k\right) \end{array}$$

where the *i* value denotes the $i^{\mathrm{th}}$ sub-band at the ${\left(j-1\right)}^{\mathrm{th}}$ level, and the number of sub-bands at the ${\left(j-1\right)}^{\mathrm{th}}$ level is equal to $i=1,\ldots ,2^{j-1}$.

## 3. Bearing Fault Diagnosis Algorithm

The algorithm for failure diagnosis presented in this study consists of two phases: the entropy features extraction phase and the classification phase. While the discriminative features extraction phase is carried out by integrating stationary wavelet packet transform and both dispersion and permutation entropy, the multi-fault classification is performed by means of a KELM model based on the Gaussian kernel function and the *k*-fold cross-validation method. We describe these phases in the next sections.

### 3.1. Proposed Diagnosis Algorithm

The steps of the bearing fault diagnosis algorithms proposed in this paper are as follows:

*Step* *1:* Divide the discrete time raw vibration signal into multiple non-overlapped signals of *N* data points.
*Step* *2:* Decompose the raw non-overlapping signals x(n),n=1,…,N into $D=2^{J}$ sub-band signals by using SWPT given as Equations (13a) and (13b).
*Step* *3:* Create a *D*-dimensional features vector based on multi-scale wavelet Shannon entropy as follows:
  (14) $$u_{k}=\left[1/E_{1},1/E_{2},\ldots ,1/E_{i},\ldots ,1/E_{D}\right]$$
  where $E_{i}$ represents one of the SWPFE/SWPPE/SWPDE values of the *i*th wavelet sub-band signal and *k* corresponds to the *k*th raw non-overlapping vibration signal.
*Step* *4:* Normalize the features matrix *Z* as follows:
  (15) $$z_{i}=\frac{u_{i}-u_{i,min}}{u_{i,max}-u_{i,min}}\;i=1,2,\ldots ,D$$
  where $z_{i}$ corresponds to the *i*th column of the features matrix *Z* and $u_{i,min}$ and $u_{i,max}$ denote the minimum value and maximum value of the $z_{i}$ vector, respectively.
*Step* *5:* Create the KELM classifier based on both the features matrix *Z* and the *k*-fold cross-validation method.

### 3.2. Kernel-ELM Classifier

In this section, we present a brief description of KELM and its main characteristics, based on our previous work on ELM [45] and KELM [21,22]. For more details on this topic, see [30,31,32,46].

The KELM classifier output is obtained as follows:

(16a) $$\begin{array}{cc} \hat{y}\left(z\right) & =\sum_{i=1}^{M}\ker\left({\tilde{z}}_{i},z\right)\beta \end{array}$$

(16b) $$\begin{array}{cc} \beta & ={\left(\frac{I}{C}+\ker\left(\tilde{z},\tilde{z}\right)\right)}^{†}Y \end{array}$$

where $\tilde{z}\in R^{D\times M}$ represents the set of input features vectors to be trained with the *M* value equal to the number of samples considered during the training phase, $z\in R^{D}$ denotes the input features vectors to be tested, *I* is the M×M identity matrix, the β values are output weights of the KELM classifier, and the *C* parameter corresponds to the regularization value. The ${\left(\cdot \right)}^{†}$ expression corresponds to the Moore–Penrose generalized inverse matrix [47]; *Y* corresponds to the desired output pattern matrix; and the function ker(·) corresponds to the Gaussian kernel given as:

(17) $$\ker\left(\tilde{z_{i}},\tilde{z_{j}}\right)=exp\left(-\frac{∥\tilde{z_{i}}-\tilde{z_{j}}{∥}^{2}}{2\sigma^{2}}\right)$$

where the σ parameter corresponds to the kernel width, which is set to $\sigma^{2}=D$ and the *D* value corresponds to the dimensionality of the input features vectors passed onto the KELM classifier during both the training and the testing phases (see Equation (14)).

Finally, the class label predicted for sample *z* is computed as follows:

(18) $$Label\left(\hat{y}\left(z\right)\right)=max\left\{{\hat{y}}_{1}\left(z\right),\ldots ,{\hat{y}}_{10}\left(z\right)\right\}$$

Using the five-fold cross-validation (CV) method [48,49], the regularization parameter, *C*, is chosen from the range $\left\{10^{1},\ldots ,10^{6}\right\}$.

### 3.3. Experimental Setup

**Figure 1:**
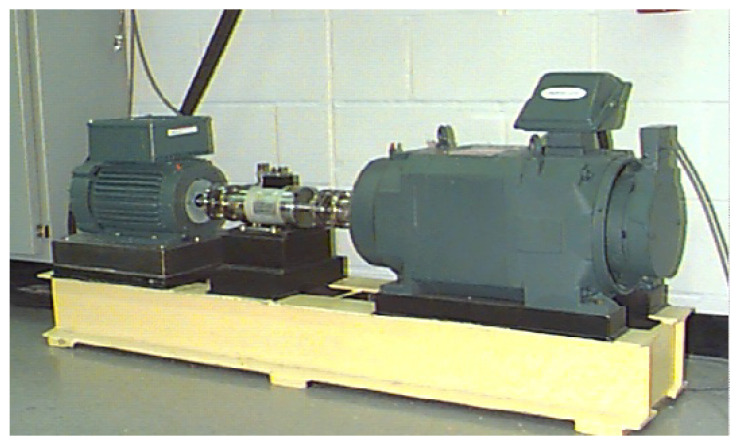
Experimental setup [33].

In this paper, we considered experimental raw vibration signals obtained from the bearing data center of the Case Western Reserve University [33], which consisted of two bearings: the drive-end (6205-2RS JEM SKF, deep groove ball bearing) and the fan-end (6203-2RS JEM SKF, deep groove ball bearing) bearings. An experimental setup as the one shown in Figure 1 was used to generate this dataset. This setup consisted of a 2-hp Reliance Electric motor, a dynamometer, and a torque transducer/encoder. The bearing holds the motor shaft during the experiments. In order to collect vibration signals, an accelerometer mounted on the motor housing, as the one shown in Figure 1, was used. Single-point failures with different failure diameters of 7, 14, and 21 mils (1 mils =0.001 inches) were introduced to both the driving-end and the fan-end bearings using the electro-discharge machining method, with the motor speed varied at 1730 r/min, 1750 r/min, 1772 r/min, and 1797 r/min for loads of 3, 2, 1, and 0 hp, respectively, leaving a total of 40 vibration signals. The length of these raw vibration signals was set to 120,000 data points (obtained in 10 s). Each of these 40 signals was divided into 50 segments. The size of each segment was set to 2400 data points (≈6-times the rotation shaft period). Table 1 shows these values.

Digital data were produced at 12,000 and 48,000 samples per second during 10 s for the fan-end and the drive-end, respectively. In both cases, data were produced for normal bearing (NB) condition samples and failure condition samples: outer race fault (ORF), inner race fault (IRF), and ball fault (BF). Further details on the experimental setup can be found in [33].

## 4. Experiments and Results

We performed experiments on the two datasets presented above. With these experiments, we aimed to evaluate the performance of the diagnostic methods proposed in this paper. To this end, first, we applied the *J*-levels SWP transform to decompose the non-overlap raw signal into $D=2^{J}$ sub-band signals. Then, for each wavelet sub-band signal, we computed the Fourier entropy value, the permutation entropy value, or the dispersion entropy value, as appropriate, using Equations (1), (6), and (11), respectively. Finally, the KELM classifier was used to diagnose the bearing fault types with different severities. Equation (16) was applied to compute the output weights of the KELM classifier, and a five-fold cross-validation method was used to adjust both the number of input features *D* and the regularization parameter *C*. To this end, the bearing vibration signal database was split into five folds, where four out of the five folds were used during the training phase and the remaining fold was used during the testing phase. To evaluate the performance of the KELM model during the testing stage, we considered the well-known average accuracy and F-score measures, which are computed as explained in [21,22,50,51].

### 4.1. Case 1: Fan-End Bearing

**Figure 2:**
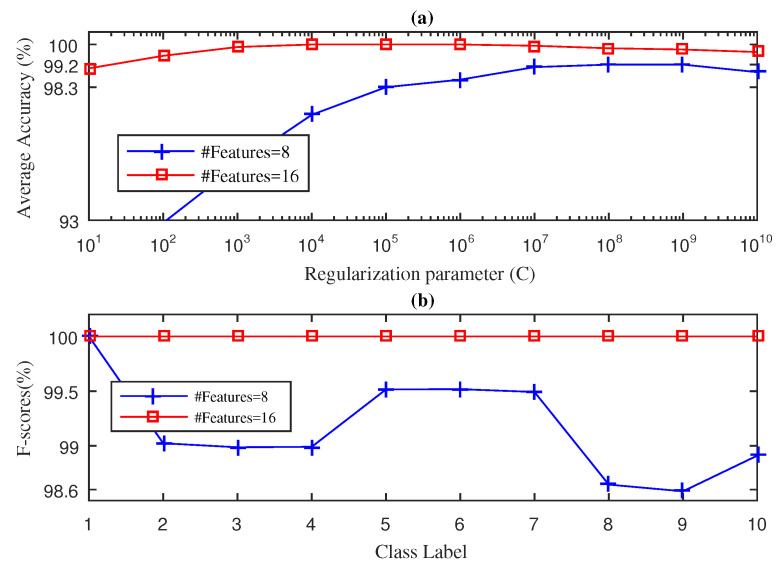
Stationary wavelet packet Fourier entropy (SWPFE)-kernel extreme learning machine (KELM) diagnosis result with five-fold CV during the testing phase for the fan-end bearing: (**a**) average accuracy and (**b**) F-score.

**Figure 3:**
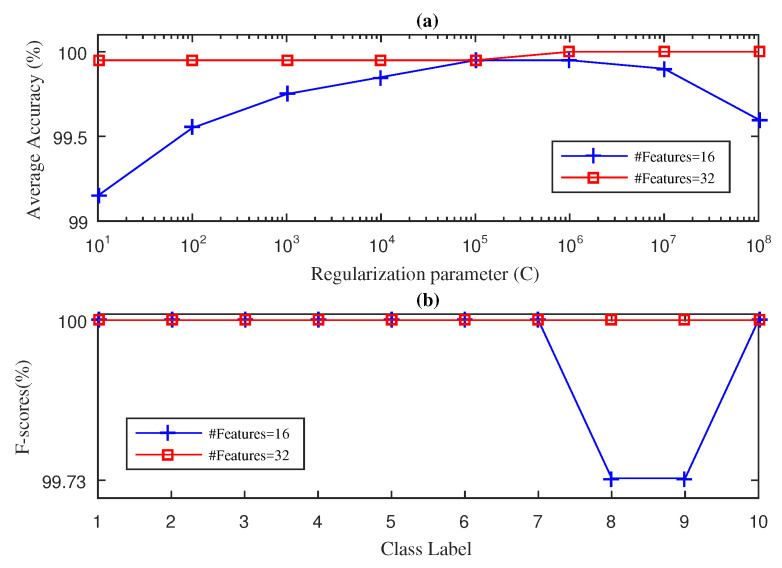
SWPPE-KELM diagnosis result with five-fold CV during the testing phase for the fan-end bearing: (**a**) average accuracy and (**b**) F-score.

**Figure 4:**
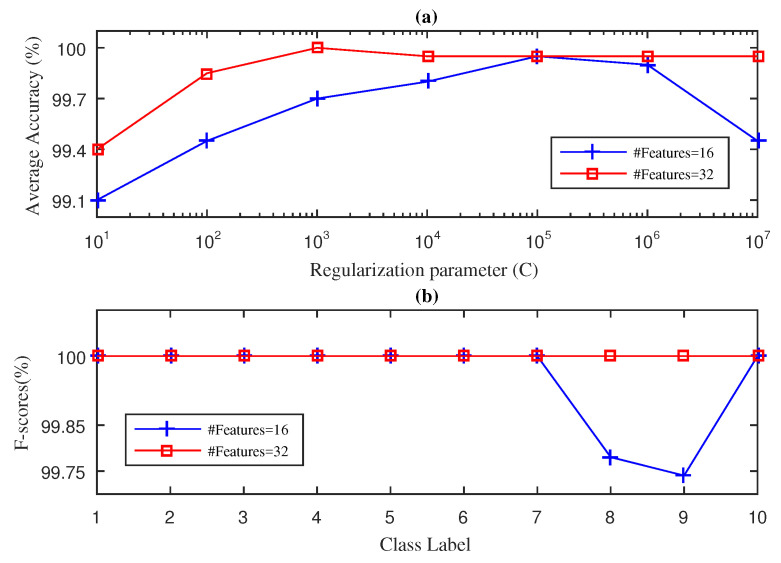
SWPDE-KELM diagnosis result with five-fold CV during the testing phase for the fan-end bearing: (**a**) average accuracy and (**b**) F-score.

In this section, we present the results obtained for the fan-end bearing when using the three approaches considered in this paper: SWPFE, SWPPE, and SWPDE. For each of these, we show both the average accuracy and the F-score measures (see Figure 2, Figure 3 and Figure 4, respectively).

For the fan-end bearing dataset we used in this paper, the collected data consisted of nine faulty bearing conditions with three failure diameters (7, 14, and 21 mils) and normal bearing condition, giving a 10-class recognition problem. For each class, there were 200 samples and a total of 2000 samples.

Figure 2 shows the results obtained by the SWPFE-KELM method. As we can see in Figure 2a, when D=8 input features were considered, the method was not able to reach 100% average accuracy, as its best obtained value was 99.20% when $C=10^{8}$. For these values (D=8 and $C=10^{8}$), we then computed the F-score for all 10 different working conditions. As we can see in Figure 2b, the SWPFE-KELM method only reached the 100% of the F-score for the NB condition.The F-score values for the remaining working conditions ranged from 98.6%–99.5%. If we increase the number of input features to D=16, the 100% of average accuracy was reached for $C=\{10^{4},10^{5},10^{6}\}$ values. We should note that, for this experiment, as the number of input features increased, the *C* value needed to reach high accuracy levels decreased. Just as we did for D=8, we computed the F-score value using D=16 and $C=\{10^{4}\}$ values. In this case, the method reached a 100% F-score value for all 10 working conditions.

Figure 3 shows the results obtained by the SWPPE-KELM method using the embedding dimension m=6 [21]. Unlike the SWPFE-KELM method, when D=16 input features were considered, 100% average accuracy was never reached by the SWPPE-KELM, and its best value (99.85%) was obtained for $C=10^{5}$. Further, for D=16 and $C=10^{5}$, the F-score reached a 100% value for all but two working conditions. It is interesting to note that the two working conditions for which the F-score was below the 100% corresponded to ball failures with 7 and 14 mils. This confirms that ball failures are harder to diagnose as reported previously in the literature [21,22]. Again, as we increase the number of input features to D=32, the SWPPE-KELM method was able to reach the 100% average accuracy value for $C=\{10^{8},10^{9},10^{10}\}$. Note that, unlike in Figure 2, increasing the number of input features did not lead to smaller values of *C* needed to reach high accuracy levels. Indeed, as the *C* value actually became larger w.r.t. the value needed for D=16, we can conclude that there was no direct relation between *D* and its *C* optimal value. We then computed the F-score value using D=32 and $C=10^{8}$. As is shown in Figure 3b, the F-score value reached 100% for all 10 working conditions.

Figure 4 shows the results obtained by the SWPDE-KELM method considering *c*, the number of states of the dispersion entropy, equal to five [21]. As we can see in Figure 4a, when D=16 input features were considered, the best accuracy value obtained by the SWPDE-KELM method was below 100% (99.92%), and it was achieved when $C=10^{5}$. We then computed the F-score value using D=16 and $C=10^{5}$. Figure 4b shows that the F-Score reached the 100% for eight out of the 10 working conditions. Again, the working conditions for which the F-score was below 100% corresponded to ball failures (7 mils and 14 mils). We then increased the number of input features to D=32, and the SWDE-KELM method was able to reach 100% average accuracy when $C=\{10^{3}\}$. The F-score value was then computed for D=32 and $C=10^{3}$. As we can see in Figure 4b, the SWPDE-KELM method reached a 100% F-score value for all 10 failure classes.

### 4.2. Case 2: Drive-End Bearing

In this section, we present the results obtained for the drive-end bearing when using the three approaches considered in this paper: SWPFE, SWPPE, and SWPDE. For each of these, we show both the average accuracy and the F-score measures (see Figure 2, Figure 3 and Figure 4, respectively). As mentioned in Section 1, we need to point out that the 48-kHz drive-end dataset used in our experiments was by far harder to diagnose than both the 12-kHz drive-end and the fan-end bearing datasets commonly used in the literature [26].

**Figure 5:**
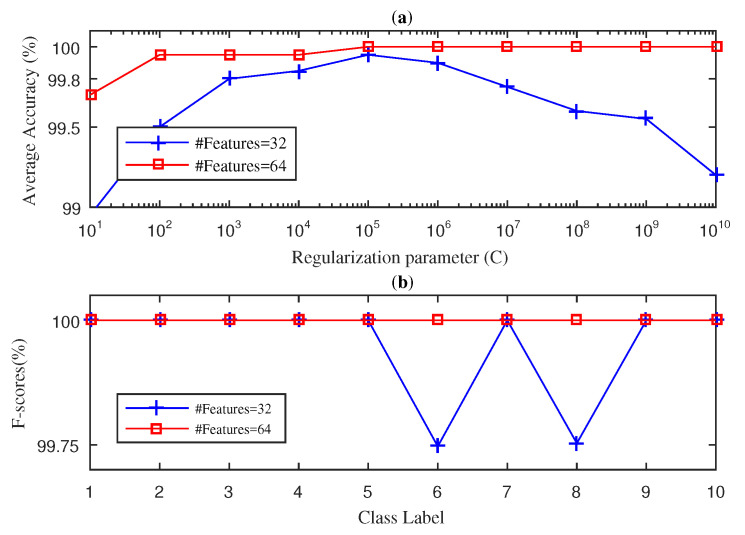
SWPFE-KELM diagnosis result with five-fold CV during the testing phase for the drive-end bearing: (**a**) average accuracy and (**b**) F-score.

Figure 5 illustrates the results obtained for our SWPFE method during the testing stage. As we can see in Figure 5a, when D=32 input features were considered, the method was not able to reach 100% average accuracy as its best obtained value was 99.95% when $C=10^{5}$. For these values (D=32 and $C=10^{5}$), we then computed the F-score for all 10 different working conditions. As we can see in Figure 2b, the SWPFE-KELM method reached a 100% F-score for all but two conditions (inner ring fault of 14 mils and ball fault of 14 mils). When we increased the number of input features to D=64, the 100% of average accuracy was reached for $C=\{10^{5},10^{6},10^{7},10^{8},10^{9},10^{10}\}$ values. Just as we did for D=32, we computed the F-score value using the D=64 and $C=\{10^{5}\}$ values. In this case, the method reached the 100% F-score value for all 10 working conditions.

**Figure 6:**
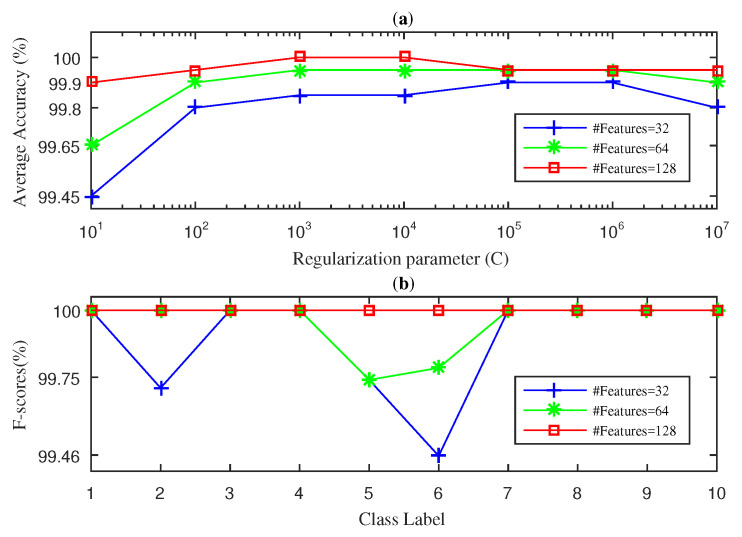
SWPPE-KELM diagnosis result with five-fold CV during the testing phase for the drive-end bearing: (**a**) average accuracy and (**b**) F-score.

We then tried the SWPPE-KELM method on the drive-end bearing signals using the embedding dimension m=6 [21]. Figure 6 shows the performance evaluation of the SWPPE-KELM method during the testing phase for the average accuracy values and the F-score values. Unlike previous experiments, here we considered three different values for the parameter *D*, the number of input features. As we can see in Figure 6a, the SWPPE-KELM method only reached an average accuracy of 100% when D=128 input features and $C=\{10^{3},10^{4}\}$. When D=32, the best obtained average accuracy value was 99.90%, and it was obtained with $C=\{10^{5},10^{6}\}$, while for the D=64 input features, the best average accuracy value (99.95%) was reached when $C=\{10^{3},10^{4},10^{5},10^{6}\}$. The F-score values obtained using our SWPPE-KELM method with D=32, D=64, and D=128 input features and $C=10^{5}$, $C=10^{3}$, and $C=10^{3}$, respectively, are shown in Figure 6b. As can be seen, the SWPPE-KELM method achieved a 100% F-score value for all working conditions only for D=128 input features. On the contrary, when fewer input features were considered, the method built with D=32 achieved an F-score value of 100% in only seven out of 10 working conditions, failing in the diagnosis of the outer ring failure (7 mils) and the inner ring failure (7 and 14 mils). Similarly, when D=64 input features were considered, the F-score value of 100% was reached in all but two working conditions: the SWPPE-KELM method failed to classify both 7 and 14 mils outer ring failures.

**Figure 7:**
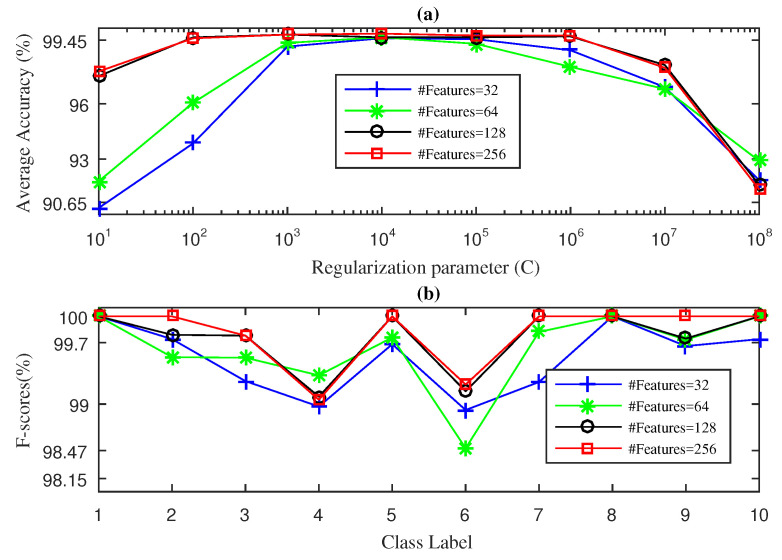
SWPDE-KELM diagnosis result with five-fold CV during the testing phase for the drive-end bearing: (**a**) average accuracy and (**b**) F-score.

Finally, Figure 7 shows the results obtained by the SWPDE-KELM method considering *c*, the number of states of the dispersion entropy, equal to five [21]. In this case, we report the results obtained for D={32,64,128,256}. As we can see in Figure 7a, the best average accuracy value obtained by the SWPDE-KELM method was 99.80%, and it was reached for D=256 using $C=\{10^{4}\}$. Similarly, for D=32 and D=64, the SWPDE-KELM method cannot even achieve the best value achieved for D=256 and D=128 as it only obtained 99.50% and 99.60% as best values, using $C=\{10^{5}\}$ and $C=10^{4}$, respectively. We then computed the F-score value using D=32 and $C=10^{4}$, D=64 and $C=10^{4}$, D=128 and $C=10^{3}$, and D=256 and $C=10^{3}$. Figure 7b shows the obtained F-score values. As we can see, the method was not able to achieve a 100% F-score value for the ORF with damage sizes of 14 mils and 21 mils and for the IRF with a damage size of 14 mils. Further, when using D=32, the method only reached the 100% value for only two out of 10 working conditions, while for D=64, the method reached the 100% value for only three working conditions. The method performed slightly better when D=128 input features were considered, reaching a 100% F-score value in five out of 10 working conditions, while for D=256, the method reached a 100% value for seven out of 10 working conditions.

Based on the results discussed above, we can say that our proposed SWPFE method has the following advantages over the SWPDE and the SWPPE methods: (i) The SWPFE method was able to achieve the 100% value for both the F-score and the average accuracy measures using only half of the failure features that the SWPDE and the SWPPE needed to reach such values. In fact, for the harder dataset (48-kHz drive-end), the SWPDE was not able to reach the 100% F-score value even though it was tested with 256 failure features (four-times the number of features needed by our proposed method). (ii) The fact that our method required fewer failure features than the SWPDE and the SWPPE methods also means that our method was faster in both the training and the testing phases. (iii) Unlike the SWPPE and the SWPDE, which require the dimension embedding hyperparameter, *m*, to be calibrated [21,36,37,38,39], the proposed SWPFE did not need any additional hyperparameters to find the entropy measure. Further, this advantage became more evident when we looked at the SWPDE method, which required the number of state hyperparameters, *c*, to be calibrated. This advantage made our method less dependent on user experience/expertise.

## 5. Conclusions

This article presented a new discriminative failure feature to improve the bearing multi-fault diagnosis in rotational machines. This failure feature integrated the SWP transform and Fourier amplitude spectrum. To this end, we considered transforming raw vibration signals from the time domain to the time-frequency domain by means of the SWP transform and, then, transforming the wavelet sub-band signals from the time frequency domain to the frequency domain by means of the discrete Fourier transform. Once the signal was in the frequency domain, we computed the failure feature, which we called stationary wavelet packet Fourier entropy.

As the main advantages of our proposed approach, we found that it needed fewer features to achieve high accuracy diagnosis w.r.t. the state-of-the-art methods previously proposed in the literature. Furthermore, our method did not require any hyperparameter calibration step, which made it less dependent on user experience/expertise.

The results clearly showed that our method outperformed both the SWPPE and the SWPDE methods in both the average accuracy and the F-score measures. This difference became more evident for the 48-kHz drive-end dataset, which is harder to solve than the commonly-used datasets in the literature.

Although only experimental data have been considered in this study, it is important to note that, as has been shown in the literature previously, diagnosis algorithms tuned using experimental data can be successfully used in more realistic environments for fan-end bearings [23].

As future work, we aim to apply the proposed SWPFE method to both bearing run-to-failure and gear-box vibration signals. Moreover, we think that comparing spectral entropy measures and well-known indicators such as spectral kurtosis, the spectral Lp/Lq norm, the spectral Gini index, or spectral smoothing might help to determine the relevance and complexity of all these measures when solving bearing multi-fault diagnosis in rotational machines. Finally, we also aim to create new bearing failure features based on synchrosqueezing time-frequency analysis to rotating machine failure diagnosis under variable working conditions.

## Figures and Tables

**Table 1:** Structure of bearing datasets. NB, normal bearing; ORF, outer race fault; IRF, inner race fault; BF, ball fault.

Fault Types	Speed (r/min)	Load (hp)	Fault Diameter (mils)	Samples Numbers	Class Label ${}^{1}$	Class Label ${}^{2}$
NB	1797-1730	0-3	0	200	1	1
ORF	1797-1730	0-3	7	200	2	2
			14	200	3	3
			21	200	4	4
IRF	1797-1730	0-3	7	200	5	5
			14	200	6	6
			21	200	7	7
BF	1797-1730	0-3	7	200	8	8
			14	200	9	9
			21	200	10	10

${}^{1}$ Fan-end bearing at 12 kHz; ${}^{2}$ drive-end bearing at 48 kHz.
